# Exploring patient and caregiver perceptions of the meaning of the patient partner role: a qualitative study

**DOI:** 10.1186/s40900-023-00511-9

**Published:** 2023-11-28

**Authors:** Anna Maria Chudyk, Roger Stoddard, Nicola McCleary, Todd A. Duhamel, Carolyn Shimmin, Serena Hickes, Pan-Canadian group of patient and public advisors, Sandra Dalziel, Delane Linkiewich, Lesley Norris, Kurt Schreiner, Kathy Smith, Janice Sumpton, Annette S. H. Schultz

**Affiliations:** 1 Department of Family Medicine, Rady Faculty of Health Sciences, University of Manitoba, CR3024 - 369 Tache Avenue, Winnipeg, MB R2H 2A6 Canada; 2 Horizon Health Network, 80 Woodbridge Street, Fredericton, New Brunswick E3B 4R3 Canada; 3 Ottawa Hospital Research Institute - Clinical Epidemiology Program, Room L1202, Box 711 - 501 Smyth Road, Ottawa, ON K1H 8L6 Canada; 4 School of Epidemiology and Public Health, University of Ottawa, 600 Peter Morand Crescent, Ottawa, ON K1G 5Z3 Canada; 5 Faculty of Kinesiology and Recreation Management, 212 Active Living Centre, Winnipeg, MB R3T 2N2 Canada; 6 Institute of Cardiovascular Sciences, St. Boniface General Hospital Albrechtsen Research Centre, R4012 - 351 Tache Ave, Winnipeg, MB R2H 2A6 Canada; 7 George and Fay Yee Centre for Healthcare Innovation, 3rd floor – 753 McDermot Avenue, Winnipeg, MB R3E 0T6 Canada; 8 Translating Emergency Knowledge for Kids (TREKK) Parent Advisory Group, Children’s Hospital Research Institute of Manitoba, 512E - 715 McDermot Avenue, Winnipeg, MB R3E 3P4 Canada; 9 College of Nursing, Rady Faculty of Health Sciences, University of Manitoba, CR3022, 369 Tache Avenue, Winnipeg, MB R2H 2A6 Canada

**Keywords:** Patient and public involvement, Stakeholder engagement, Patient engagement, Patient engagement in research, Patient-oriented research, Qualitative interviews

## Abstract

**Background:**

The re-conceptualization of patients’ and caregivers’ roles in research from study participants to co-researchers (“patient partners”) has led to growing pains within and outside the research community, such as how to effectively engage patients in research and as part of interdisciplinary teams. To support the growth of more successful research partnerships by developing a shared understanding of how patient partners conceptualize and contribute to their role, this study aimed to explore patient partners’ motivations for engagement and understanding of their role.

**Methods:**

We conducted semi-structured interviews with participants (*n* = 13) of an online survey of activities and impacts of patient engagement in Strategy for Patient-Oriented Research projects. Eligibility criteria included being a patient partner that indicated interest in interview participation upon survey completion, the ability to read/write in English and provide informed consent. Data were analyzed thematically using an inductive, codebook thematic analysis.

**Results:**

Illuminating the lived/living patient and caregiver experience was central to how most patient partners conceptualized the role in terms of its definition, purpose, value, and responsibilities. Participants also identified four additional categories of motivations for becoming a patient partner and contributions that patient partners make to research that build upon and are in addition to sharing their lived/living experiences. Lastly, participants highlighted important connotations of the term patient partner, including temporal and context-specific considerations for the term “patient” and what “partner” may imply about the nature of the research relationship.

**Conclusions:**

At the onset of partnership, academic researchers and patient partners must create the space necessary to discuss and understand each other’s underlying motivations for partnering and their perspectives on the purpose, value, and responsibilities of the patient partner role. These early conversations should help unearth what research partners hope to get out of and feel that they are able to contribute to engaging, and in such contribute to the development of reciprocal relationships that work towards shared and valued goals.

*Trial registration* Not applicable.

**Supplementary Information:**

The online version contains supplementary material available at 10.1186/s40900-023-00511-9.

## Abstract (plain language summary)

Developing a shared understanding and respect for each other’s motivations, experiences, and expectations is an important step toward successful academic researcher-patient partnerships. Therefore, this study aimed to explore patient partners’ motivations for engagement and understanding of their role. We met this aim by interviewing 13 English-speaking individuals with lived/living experience of being patient partners on Strategy for Patient-Oriented Research projects. These individuals were identified through our database of previous study participants interested in future research opportunities. We analyzed the information gathered through interviews by identifying themes that arose among sets of interview questions. Our findings revealed that illuminating (i.e., sharing and drawing attention to) the lived/living patient and caregiver experience was central to how most participants defined the patient partner role and its purpose, value, and responsibilities. We also identified four other categories of motivations for becoming a patient partner and contributions that patient partners make to research other than sharing their lived/living experiences. Finally, interviews highlighted different types of meanings that could be attached to the title of “patient partner.” Based on our findings, we suggest that it is very important that academic researchers and patient partners take the time to discuss and understand each other’s underlying motivations for partnering and their thoughts on the purpose, value, and responsibilities of the patient partner role. These early conversations should help unearth what research partners hope to get out of and feel that they can contribute to engaging and, through this, help establish two-way relationships focused on shared and valued goals.

**Supplementary Information:**

The online version contains supplementary material available at 10.1186/s40900-023-00511-9.

## Background

The Canadian Institutes of Health Research (CIHR) states that health researchers aim to increase “… knowledge of health, disease, and health services, and to then apply that knowledge to help people lead healthier lives” [1]. Patients and their unpaid caregivers (e.g., family or friends) have direct [lived/living] experience of health, illness, and accessing health care services. Thus, they are well positioned to be important allies in researchers’ pursuit of health-related knowledge and its applications to wellness and the betterment of the healthcare system. Despite significant investments in initiatives such as CIHR’s Strategy for Patient-Oriented Research (SPOR) and the Patient-Centered Outcomes Research Institute in the United States, patients’ and caregivers’ contributions to health research have been, for the most part, underutilized and limited to the role of research subject. While there is no doubt that without human participants, much of health research would not function, with the development of these initiatives came formal acknowledgment of the other significant roles that patients and caregivers can and *should* play in health research. Namely, as co-researchers (“patient partners”), helping to drive research processes and ensuring a focus on patient and caregiver-identified priorities and outcomes [2] – ideally through engagement from the outset of the study (when ideas are being generated and the research question defined) and into knowledge translation and dissemination. Within Canada, this patient-centered research approach is referred to as patient engagement in research.

As with any change, the re-conceptualization of the patient’s and caregiver’s role in research has led to growing pains within and outside the health research community. Specifically, one notable challenge pertains to teamwork among interdisciplinary and cross-disciplinary teams [3–5], which is compounded by the fact that many patients and caregivers are external and likely new to the research environment. As well, research suggests that many academic researchers are uncertain about how to effectively include patient partners in the research team [6, 7]. Careful consideration of how research collaborations are developed and maintained with patient partners is imperative to successful teamwork (e.g., engage a diversity of perspectives, obtain meaningful input, achieve desired outcomes). One defining characteristic of teamwork is collective cooperation toward shared and valued goals [8]. Thus, successful research collaborations require team members to learn how to work together while representing their own perspective as they work to co-create and achieve a shared vision. To this end, an important step towards developing and maintaining successful academic researcher-patient partnerships is a shared understanding of, and respect for, the motivations and expectations of different partners and the unique experiences, skillsets, and knowledge they contribute to reaching the team’s goals.

## Methods

### Aim

In this study–which was co-designed, co-led, and co-authored with a patient partner and engaged other patient partners as members of the research team and as advisors–we explored the experiences of patient partners. Specifically, we aimed to explore patient partners’ motivations for engaging in research and their understanding of the role. Our underlying research question was, “What motivates patient partners to engage in research and what meanings do they attach to the role?” Our study adds data from the Canadian context to the growing international body of literature examining patient partners’ motivations and role expectations for engagement [9–15]. Novelly, not only did we engage patient partners across this study’s research cycle, we also involved study participants (all of whom represented the patient partner perspective) in helping guide its analysis and write-up. Creating this space for patient partners to share their ideas and experiences of engagement is offered as a mechanism to shape shared understandings of patient engagement in research and help disrupt the dominance of the academic researcher voice in patient engagement and health research. Also unique to our work is the creation of an accompanying lay version of our paper (Additional file 1) that aims to broaden the reach of our findings to a non-academic audience, and an editable document (Additional file 2) aimed at guiding academic researchers and patient partners in directly applying our findings to their own partnerships. As such, our findings should help enrich the understanding of how patient partners conceptualize and contribute to their role and more directly support the development of more meaningful relationships between academic researchers and patient partners.

### Study design and setting

This qualitative study was part two of a sequential explanatory mixed-methods project (i.e., cross-sectional survey [16], followed by semi-structured interviews (current study), and finally, a national workshop (under review)) that aimed to describe the enactment of patient engagement in projects funded through SPOR initiatives and identify future directions for the field. The research team included two patient partners (RS, SH), a patient-oriented researcher (AMC), two senior scientists (ASHS, TAD), an implementation scientist (NMC), and a patient engagement specialist (CS). The conceptualization for this current study emerged from a patient partner (RS), and the intention was to explore the experiences of patient partners involved in SPOR-funded research [17]. Thus, this study was guided by a constructivist standpoint where the research team endorsed a relativist ontology (i.e., all people experience a different reality; each patient partner has their own perception of being engaged in research) and a subjectivist epistemology (i.e., knowledge is co-created; academic researchers, patient partners, and research participants will co-create knowledge in the present study) [18, 19]. Therefore, it was important for us to center the voices of patient partners when co-generating knowledge through this study. The Guidance for Reporting Involvement of Patients and the Public (GRIPP-2) short-form checklist informed our reporting (Additional file 3) [20]. Ethics approval was obtained from the Education Nursing Research Ethics Board at the University of Manitoba (certificate number E2019:082(HS23180)).

### Sample and recruitment

In 2020, we conducted a Pan-Canadian cross-sectional survey of patient engagement activities and impacts of SPOR-funded projects [16]. At the end of the survey, we invited respondents that represented the patient partner perspective to indicate if they were interested in participating in the current qualitative interview study. The lead author (AMC) contacted the 15 respondents who expressed interest in participating in interviews via an email that contained a study overview and consent form. As interviews were conducted virtually, participation was not limited to any specific province. The lead author then followed up with those who confirmed interest in study participation to ensure they met study eligibility criteria and to answer any of their questions. In addition to being a patient partner that completed our cross-sectional survey and indicated interest in participating in subsequent qualitative interviews, individuals needed to be able to read and or write in English and provide written informed consent to be eligible to participate. Individuals were compensated $75 for participating in the study (interview and member check activities).

### Data collection and tools

Semi-structured interviews were co-conducted virtually (via Microsoft Teams) by a patient partner (RS) and academic researcher (AMC). Three of the co-authors (AMC, ASHS, RS) co-developed a semi-structured interview guide, as guided by the literature, preliminary cross-sectional survey findings, and their collective experiences with patient engagement in research, and then shared with the rest of the research team for feedback. Two co-authors (AMC and RS) piloted the interview guide with four individuals with experience of being patient partners (none of whom were eligible to participate in the study) to further refine its content, including editing the order and structure of the questions for clarity. Study interviews took place February–April 2021, were recorded, and lasted approximately 60–90 min each. They began with an overview of the study, which was followed by a set of closed-ended sociodemographic questions, and then moved into a series of open-ended questions [Additional file 4]. These open-ended questions probed participants’: (a) reasons for becoming a patient partner; and their perceptions of (b) how to define the term patient partner, (c) the purpose and/or value of patient partners, and (d) the roles and/or responsibilities of patient partners in research. Following the interviews, recordings were sent to a professional service for transcription. All participants provided written, informed consent prior to study participation.

### Codebook thematic analysis

We implemented an inductive codebook thematic analysis as recently described by Braun and Clarke to analyze the qualitative dataset (i.e., interview transcripts) [21]. This method of analysis supported our aim to explore patient partners’ motivations for engaging in research and their understanding of the role. Codebook thematic analysis aligns with our constructivist approach that positions data analysis as a subjective practice [21], but is more pragmatic than reflexive thematic analysis as it can accommodate multiple researchers working together on the analysis in a more efficient timeline. Thus, codebook thematic analysis is an optimal method when engaging patient partners with less time and less research training in a qualitative data analysis [21]. The extent to which RS and SH were engaged in data analysis was decided through discussion and based upon their preferences for involvement in the study.

Anonymized data (i.e., interview transcripts) were uploaded into NVivo Version 12 (QSR International). As our intention was to listen to the voices of patient partners, our analysis process aimed to maximize input from our patient partner team members and those who participated in an interview [17, 18]. Our approach to thematic analysis began with three study team members (AMC, ASHS, RS) independently reviewing two randomly chosen interview transcripts, and inductively generating plausible codes under each set of interview questions. They then met to discuss and agree upon a codebook used to conduct the remainder of the analysis. The codebook was iteratively refined by the lead author (AMC) while coding the remaining transcripts. When alternate plausible codes were generated, the lead author (AMC) consulted with the other two co-authors co-leading the initial analysis (RS, ASHS). Once a preliminary analysis of all the transcriptions was completed, the lead author (AMC) sorted the codes and their corresponding data excerpts into potential sub-themes under each of the four main interview questions (themes) that were used to explore participants’ motivations for becoming patient partners and their understanding of the role. The lead author (AMC) discussed the development of sub-themes with RS and ASHS until agreement on interpretation was achieved. A list of themes, sub-themes, and corresponding codes and data excerpts was shared with the entire study team for feedback and refinement. Next the revised themes, sub-themes and corresponding codes and data excerpts were shared with all study participants, who were invited to help member-check the accuracy of and build upon the findings during focus groups held August–September 2021.

A paper describing our interactive approach to engaging participants in member-check activities will be published as a stand-alone paper. Briefly, the member-check activities were informed by our research team’s previous qualitative research and patient engagement experiences and conceptualized by the first and senior authors (AMC, ASHS) and RS. Participants were first sent a fillable pdf document that contained the revised themes, sub-themes and corresponding codes and data excerpts. For each set of respective sub-themes, codes, and data excerpts, the document instructed participants to describe whether: they saw themselves reflected in was presented; there was anything missing or that they would like to add; anything stood out; or they had any other comments they would like to make. Participants had the option of meeting with the first author virtually if they preferred to give verbal responses and all questions were optional. Participants each then attended one follow-up member-check focus group (*n* = 2 were held to accommodate everyone’s schedule) to discuss their feedback (so as to arrive at a final set of themes, sub-themes, and excerpts) and what they viewed as the most important findings. All participants took part in the member-check activities and were provided with an individualized participant report at the end of the study that summarized how we incorporated their input in the activities into shaping the study’s findings.

### Patient engagement in the study

This study relied on emails, phone calls, and small and full team meetings to engage two patient partners across its research cycle at the levels of collaborate (RS) and involve (SH) [22, 23]. Specifically, RS co-led the study, meaning he played an active role in designing and executing all of its activities and had an equal say in its decision-making (along with academic researcher co-leads AMC and ASHS). SH (along with the remaining members of the research team – CS, NMC, TAD) was engaged at major milestones throughout the study (e.g., grant writing, study planning, data analysis and synthesis) to help guide and elicit feedback on study directions, materials, and outputs, and to inform study-related decisions that were ultimately made by the study co-leads. The study also consulted [22, 23] participants to help ensure the accuracy, and make sense of, its findings and write-up through member-check focus groups and sharing study documents to gather written and verbal feedback. Both RS and SH co-authored this article, as did study participants that engaged in the consultation activities and expressed written interest in group authorship. Individuals that chose to contribute to the group authorship were given the option to not have their name included on the authorship list to preserve their participant confidentiality and were only presented with anonymized data as presented in the member-check focus groups. To help support patient partners’ co-authorship, we provided a lay summary of the paper (similar to Additional file 1) along with the paper formatted for submission to a journal and gave individuals the option of meeting with the first author virtually to go through the paper and discuss revisions together. Although there are no concrete next steps for engaging this group of patient partners in follow-up research, all were asked whether they would like to be notified by the team about future research and/or patient engagement related opportunities.

## Results

Of the 15 individuals contacted, 13 agreed to participate. Table 1 presents participants’ select self-reported sociodemographic characteristics.

**Table 1:**
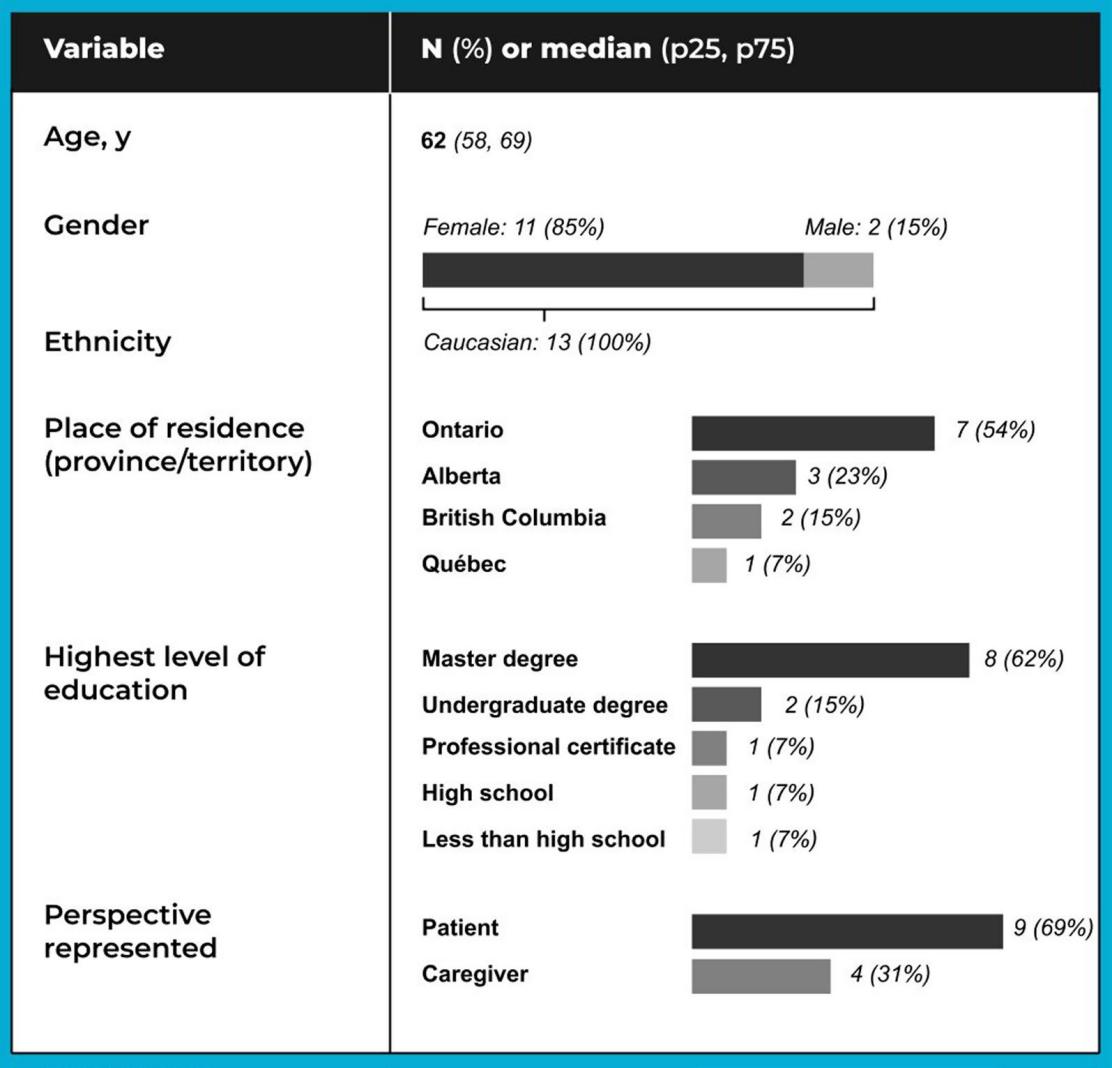
Participants’ select sociodemographic characteristics (*n* = 13)

**Fig. 1:**
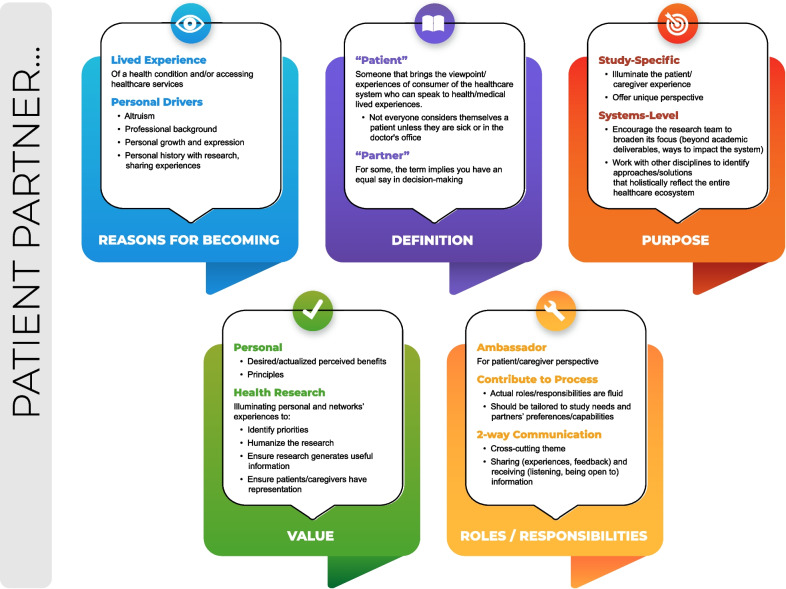
Summary of study findings

As summarized in Fig. 1, we next present a summary of findings generated from the interview data. The four main interview questions that were used to explore participants’ motivations for becoming patient partners and their understanding of the role are presented as overarching themes. Subthemes are used to provide topic summaries of patient partners’ responses to each question. Presenting qualitative findings as summaries of responses to interview questions is consistent with codebook thematic analysis [24]. We have assigned pseudonyms to participant quotes, including pseudonyms that could be considered gender-neutral, to preserve participant anonymity within the SPOR-funded patient partner community and to also contribute to the humanizing of patients engaged in research. A simpler and more accessible overview of our findings, and their applications, is found in Additional file 1.

### Reasons for becoming a patient partner

**Fig. 2:**
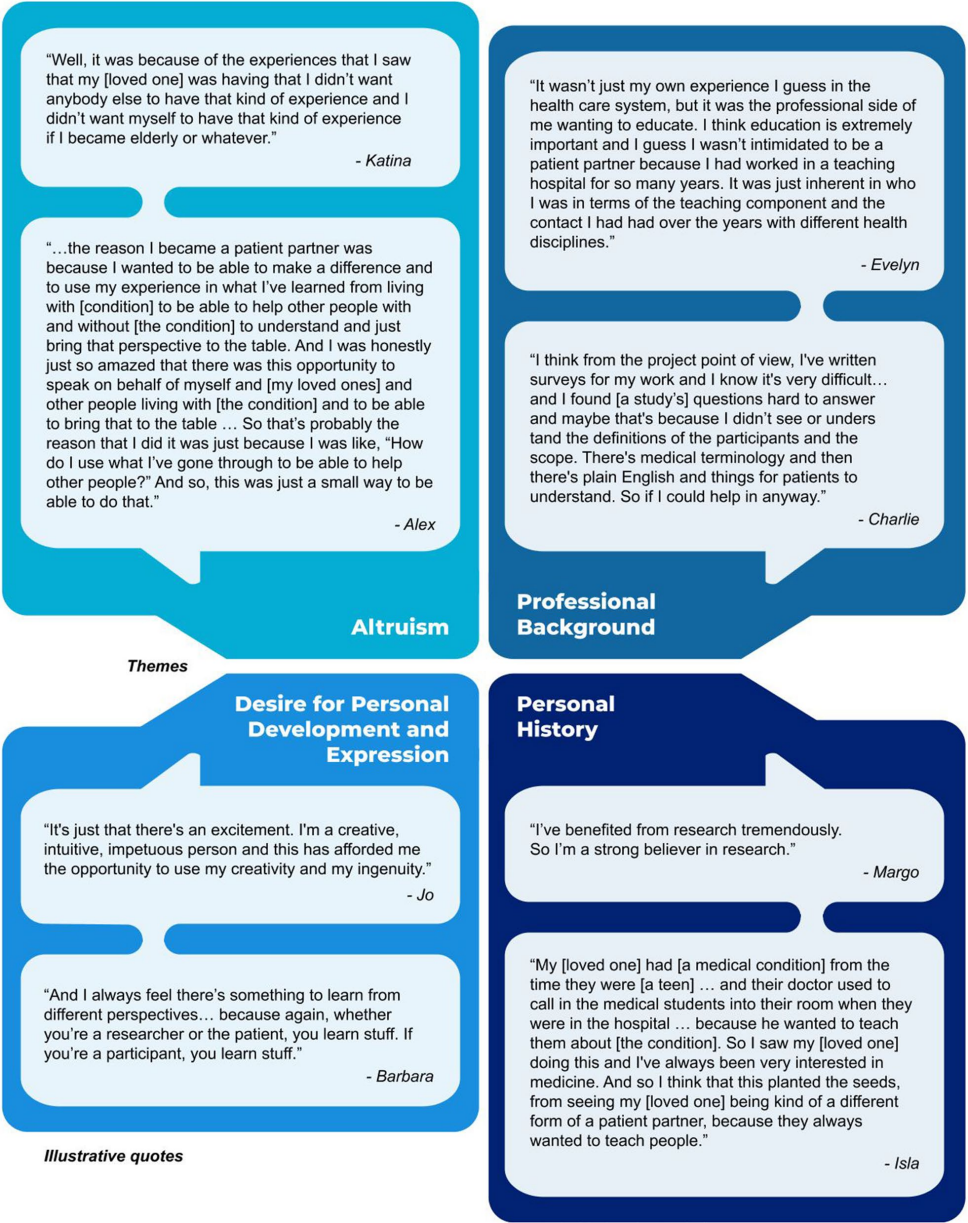
Illustrative quotes for reasons for becoming a patient partner

Participants’ experiences of a health condition and/or accessing health care services provided them with the prerequisite lived/living experience needed to be a patient partner. However, as illustrated by the select quotes presented in Fig. 2, they also had personal drivers that motivated them to engage in research and influenced what they hoped to achieve from partnering. These included altruism, professional background, desire for personal growth and expression, and personal history.

### Altruism

Participants’ experiences with the complexities involved in navigating the healthcare system and with their or their loved one’s conditions made them want to apply their learnings to the benefit and learning of others navigating similar waters. Others spoke of a more general desire to “give back” and devote themselves to tasks greater than themselves, like breaking down silos between the research and healthcare systems and raising awareness of what it is like living with a health condition.

### Professional background

Participants’ professional backgrounds, be it in healthcare, academia, research, or creating surveys, contributed to their wanting to engage in research since they felt that they had the education, skills, and/or experience to meaningfully contribute and/or they saw partnering as a natural extension of their professional roles. These professional backgrounds were also related to interests they had for the research, such as the opportunity to be involved in asking and solving questions, and shaping projects at the formative level.

### Desire for personal development and expression

Participants also discussed viewing engagement as an opportunity for personal growth and development. For example, some stated that they were interested in engaging and challenging themselves cognitively, especially after retirement, and learning new things, including from different perspectives and other patients’ experiences. Others identified that they saw partnering as an opportunity to apply their creativity and ingenuity, expand their social and professional circle, and feel good about themselves through their involvement. One participant discussed how their motivations evolved over time. That is, they initially became a patient partner for the opportunity to connect with the healthcare system and hopefully improve their condition. Then, with time, their motivation shifted to focus more on helping others through the role.

### Personal history

Some participants stated that their own personal history of being involved as a research participant, being asked to share their lived/living experiences of a health condition to different groups of medical professionals and students and pharmaceutical representatives, and/or growing up seeing their parent being asked to share their healthcare experiences with medical students piqued their interest in research and led to opportunities to become patient partners.

### Definition of patient partner

In defining the term patient partner, participants spoke of the meaning they attached to each individual word as well as of both words considered together. What also arose was a sort of divide between participants as to whether they liked the term. Specifically, some participants’ definitions of the term patient partner focused on the viewpoint or experiences they brought to the table–be it a client or consumer of the healthcare system or of someone that could speak to their own or their loved ones’ health/medical lived/living experiences. Others took this one step further to also consider the connotations of the word partner within the context of research. To them, this could mean someone who not only had a viewpoint, but a viewpoint that was heard by the other members of the research team and that had a meaningful say in the decision-making that was occurring, ideally across the entire research cycle. As summarized by one participant:

“I define the patient partner role as one of equality and inclusion all along the entire continuum of a research process. A ‘partner’ role means you are there at the initial concept planning and decision-making stage of a project. Otherwise, you are technically only an advisor to someone else’s research. This difference needs much more defining and refining with researchers. They do not appreciate our role as such.” Jess.

Some participants also highlighted the context-specific and temporal dimension of the term patient. These participants only considered themselves a patient when at the doctor's office or having a test done or did not really consider themselves to be a patient anymore due to recovering from the condition responsible for their lived/living experience. These participants felt that perhaps the terms “lay person”, “advisor,” “consultant”, “person with lived/living experience” were more appropriate than patient partner.

“I had [a condition]… and then I became cured… and I was so grateful that when I was approached asking if I’d like to become involved in some medical research I said, yeah, I’ll try and give back if I can… I did not consider myself a patient at the time of introduction to the study, but I guess I was.” Spinner.

### Purpose of being a patient partner

Participants’ perceptions of the purpose of patient partners resided at the study and systems levels.

#### Study level

According to study participants, a fundamental purpose of patient partners is to illuminate (i.e., share and draw attention to) what is important to persons living with, or caring for someone living with, a health condition. To this end, some patient partners also have deep-rooted networks of other patients and caregivers that they can draw on to ensure that their perspectives also reflect the experiences of the broader community. Ideally, this perspective is what drives the research. However, as illustrated by the quote below, for this to occur, lived/living experience needs to be valued and recognized as a valid form of expertise by the other members of the research team.

“Just the ability to be able to use lived experience, lived expertise, and bring that, and also have it be recognized – not just bringing it to the table but having it be recognized as expertise.” Alex.

The perspectives that patient partners bring also serve other purposes, such as the contribution of practical ideas and answers to strategizing research problems, pushing academic researchers to consider and explain what the research is going to lead to and how it will ultimately affect patient lives, giving the study more credibility, and enhancing patient, caregiver, and patient partner experiences with research. As explained by a participant:

“Being a patient partner brings a completely different perspective to the research question. I know that the study that I chose involved a patient population. I know some of them were basic science studies, and it’s challenging I think to be a patient partner to a basic science study, but I think it’s still very important. Even if one doesn’t understand all of the biology that’s being looked at, I think it’s important to have a patient perspective and have that researcher be able to – kind of turn the tables on the researcher and have them be able to explain what their research is going to lead to. What their hypothesis is, what – you know, it may be very basic science right now but it might be the beginning of a new mechanism and, once we know a mechanism, then one can start looking at interventions or drug therapies for it.” Isla.

#### Systems level

According to study participants, another key purpose of patient partners is to ensure that the focus of the underlying research is not only the production of academic deliverable such as manuscripts, but also the application of research findings to enhance patient and caregiver experiences and outcomes within the healthcare system. This includes pushing the research team to use a broader lens to explore the potential avenues through which the study can impact the system and the system’s impact on patient outcomes. It also includes melding with the perspectives of everyone at the research table so as to holistically reflect the realities, experiences, and possibilities of the large healthcare ecosystem and come up with approaches and solutions that are only possible through this type of synergy.

“Many clinicians/researchers seem to have too narrow a focus on a finite project with a finite deliverable—publish a paper. I see lots of lost opportunities to use a broader lens exploring the ripples: how does a study impact the system, and how does the system impact patient outcomes.” John.

#### Value of being a patient partner

Participants’ discussion of the value of patient partners focused on the personal values that they derived from and brought to the role, as well and the overall value of patient partners to health research.

### Personal value(s)

These reflect the personal value that being a patient partner brought to participants’ lives, which may be related to their reasons for becoming patient partners discussed above (e.g., desire for personal growth, altruism). As explained by one participant:

“This is something that keeps me engaged mentally and I get a great deal of satisfaction out of doing it. So that's the sort of thing that I'm involved with.” Jo.

Personal values also reflect the principles (e.g., respect, mutual learning, authenticity) that patient partners bring to the role and shape their expectations for how they and others should be treated and the nature of engagement activities. As stated by another participant:

“If I’m playing that role of bringing other patients to the table, my values would be that they’re brought to the table in a respectful, meaningful, you know not tokenistic way and that they have all the tools that they need to participate as partners in that research. That was of vital importance to me. So everything that went to them came through us we saw every piece of correspondence, every document. Everything came through us, either written by us or with feedback from us.” Margo.

### Value to health research

According to participants, a major value of patient partners to health research stems from illuminating their personal and networks’ experiences of being a patient and/or caregiver. This sharing of experiences is important because it gives others an idea of what is important to a person, or the caregiver of a person, living with a condition or accessing healthcare services and may in turn affect research directions and outcomes in many ways. For example, this information may lead the research team to expand their focus beyond their initial conceptualization of the research problem by presenting novel ideas or perspectives to the framing of the research questions, as well as influence the direction of the research by bringing forward other questions and ideas that the research team may not have thought of on their own. Of course, in order for this to occur patient partners need to be engaged early in the study’s research cycle. Furthermore, this giving voice also helps humanize the research through sharing personal stories (that move the research from abstract scientific concepts to the experiences of someone in front of you) and contributing pragmatic and practical perspectives to scientific points of view. Downstream, this giving voice will also hopefully help ensure that the research will generate information that is important to a person, or a caregiver of a person, living with the condition. Lastly, as illustrated by the quote below, even if members of the research team have shared some similar healthcare experiences, they may not necessarily be able to bring them into the research to the same degree as patient partners because that’s not their primary focus:

“Well, when I first started in this type of role, I was questioning what kind of value I could bring, because many of the clinicians are also parents and caregivers themselves…. What I’ve learned … is that, yeah, some of them might have the same lived experience as a parent and as a caregiver, but for some reason, they’re not able to incorporate that into their work. And that’s been a big, big surprise to me… I’m reminding these folks of things from that patient perspective that they’ve probably already lived themselves, but they’re not incorporating … so, that’s one of the values – reminding them of some of those lenses and some of the input that they themselves might have given if that was their focus in the moment.” John.

### Roles and/or responsibilities of patient partners

#### Ambassador for the patient and caregiver perspective

Participants generally stated that, first and foremost, the role and responsibility of patient partners was to be an ambassador for the patient and caregiver perspective during the research process and other related interactions (e.g., with health service providers, students). This includes representing the patient and caregiver voice at the interdisciplinary research table while keeping in mind that the views expressed should not reflect personal agendas. To this end, some participants also spoke of the importance of drawing on networks to gather the feedback and perspectives of the broader patient/caregiver community, including voices traditionally less heard in research. Lastly, participants also spoke of the need to ensure that the voices of other patient partners, and patients and caregivers in general, were being respected and were informing research activities, such as engagement advisory committees they were co-leading or others’ research proposals that they were being asked to review as part of committee work.

“… I’ve been a researcher, but I’ve never stopped to think about it from the perspective of a patient to the depth that I did wearing the patient’s hat... And you don’t think of it in that depth if you’re not the patient. Because it’s you who’s going to be affected, not the people collecting the information.” Barbara

#### Contribute to the research process

**Fig. 3:**
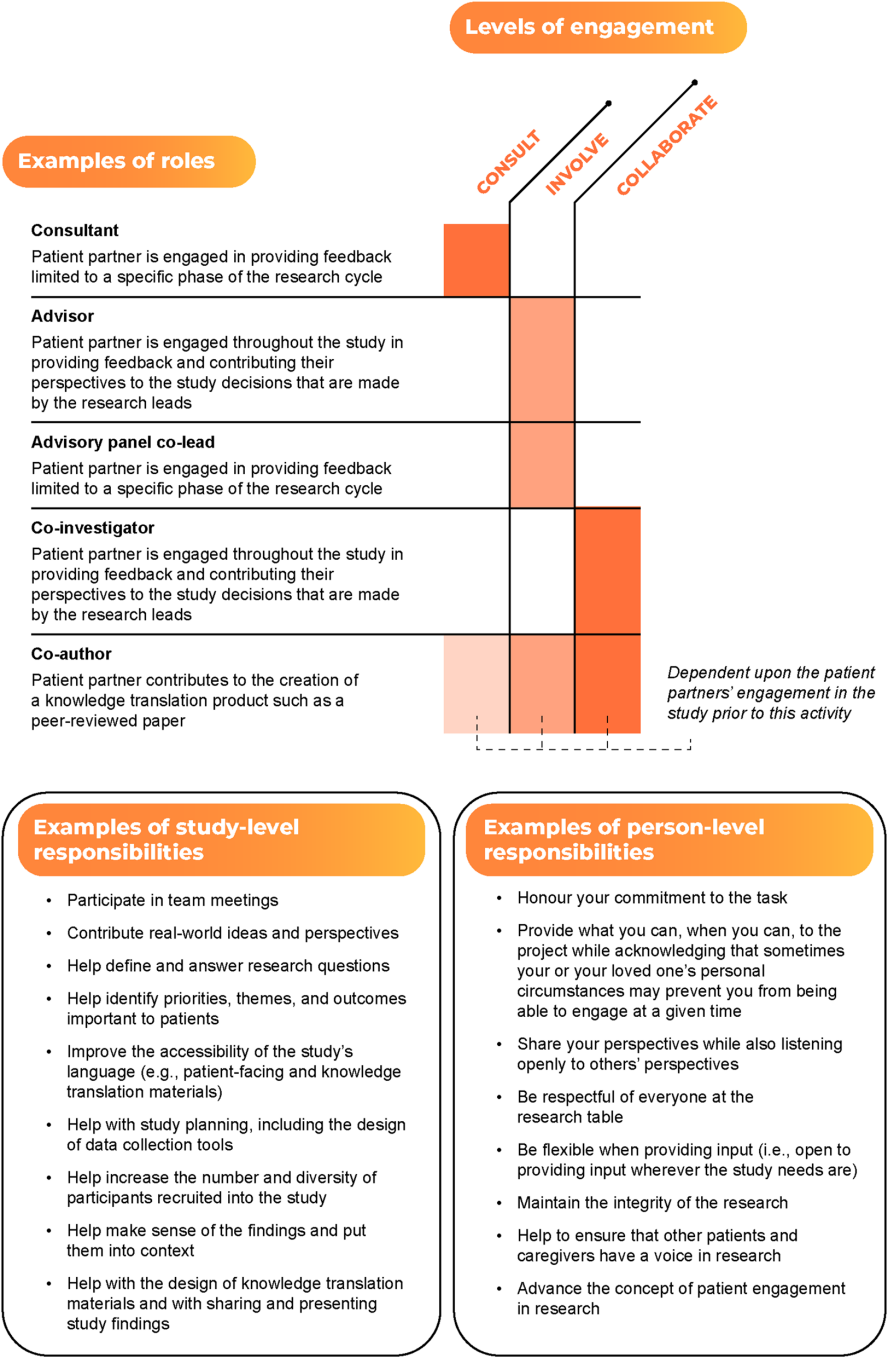
Examples of patient partner roles and responsibilities in contributing to the research process

Participants also stated patient partners’ roles and responsibilities included contributing to the research process. The specifics of how they did so, however, were “fluid,” meaning they varied across studies and patient partners. Figure 3 summarizes the provided examples of patient partner roles and responsibilities in contributing to the research process.

As expressed in the quote below, participants generally acknowledged that this variability in how patient partners contribute to the research process was due to the fact that there is no “one size fits all” when it comes to engagement. Rather, the engagement approach should be tailored to meet the study’s needs and goals, and the roles and responsibilities that patient partners are comfortable with and capable of vary. They also shared differing experiences with how their roles and responsibilities were assigned. Some participants stated these were decided through conversation and relationship building with the co-investigator and/or person overseeing engagement, with some even sharing that the way in which they contributed to the research process evolved over time and hand-in-hand with their familiarity with the project and the depth of their relationship with the principal investigator. Conversely, other participants stated that (in their experience) how patient partners could contribute to the research process was largely dictated and decided upon by the academic researcher members of the team.

“My responsibility is to represent people living with [my condition], because that’s my expertise. And my role depends very much on the project. Sometimes it’s to review the literature, the manuscript, sometimes it’s to collect data, sometimes it’s – it’s to do whatever the role is that’s decided upon once you become a patient partner.” Emma.

#### Two-way communication (i.e., speaking and listening)

In discussing roles and responsibilities, a cross-cutting theme (i.e., pertaining to both being an ambassador for the patient/caregiver perspective and contributing to the research process) that emerged focused on two-way communication between patient partners and the research team. Specifically, participants stated that patient partners were responsible for sharing their healthcare experiences openly with the research team and potentially in other related scenarios (e.g., presentations, stakeholder meetings) to the extent they are comfortable. It was also important they provided honest, objective, and constructive feedback to the research team that was specific to the research, as well as the general engagement process and other factors relevant to the partnership. Lastly, open communication (including pertaining to needs) was also identified as a key responsibility of patient partners. According to some participants, this was best supported through the identification of a designated point-person (e.g., patient engagement facilitator) that patient partners could speak about sensitive (and any other) matters.

“So I think one of them is open communication – you make the researcher aware of your needs. It’s really helpful if the team assigns one person to you so that you don’t have to be bringing your concerns to a full meeting, you know that you can just address one person. And then if you have to bring it to the meeting you do, but sometimes it’s just something you wish to discuss with somebody… It might be the PI [principal investigator], it might be one of the other co-PIs. It doesn’t have to be the person in charge, but knowing that you’ve got someone kind of has your back and that works really well.” Margo.

On the flip side of the coin, participants also discussed the importance of listening, being open to and respectful of others’ perspectives, and realizing that their viewpoint was not the only one at the research table. This opened the space for patient partner perspectives to interact synchronistically with the other perspectives at the research table to enhance research directions and processes. In support of this, another role and responsibility of patient partners is to engage in mutually respectful discussions that help to build a more dynamic research team, break down walls between patient partners and the other members of the research team, and raise the profile of patient partners through true partnership with academic researchers.

“Well, I believe we need to present our experience and our perspectives, but be respective as we discuss around the table. My responsibility is to be true to my experience and which I am and not be fearful to share it … you know, you’re the expert… so, don’t ever worry about what you say because it’s your experience and no one else’s and so not to be nervous or anything. But also you need to understand that it’s not just about you and your experience. It’s about the system or trying to incorporate what is best for everybody. But in order to gain that understanding you as a patient also need to hear other perspectives.” Jo.

## Discussion

To further the conversation on establishing and maintaining successful patient-academic researcher partnerships, this study examined patient partners’ underlying motivations for engaging and their understanding of their role in research. We found that illuminating (i.e., sharing and drawing attention to) the lived/living patient and caregiver experience was central to how most patient partners conceptualized the role in terms of its definition, purpose, value, and responsibilities. In addition, we also identified other personal drivers of becoming a patient partner, and contributions that patient partners make to research that build upon and are in addition to sharing their lived/living experiences. Lastly, our findings highlight important connotations of the term patient partner, including temporal and context-specific considerations related to the term “patient” and what the term “partner” may imply about the nature of the patient partner-academic researcher relationship. Additional file 2 applies these study findings to guiding academic researchers and patients partners in conversations aimed at arriving in a shared understanding of each others’ personal motivations for engagement and understanding of the patient partner role.

Each patient partner is a unique individual, with their own personal motivations for engaging in research. In this study, we found that patient partners’ reasons for engaging could be organized into four broad categories–altruism, their professional backgrounds, desire for personal growth and expression, and personal history with research or sharing experiences. This is similar to the findings of other studies [9–15], which collectively identified social and personal categories of reasons underlying patient partners’ desires to engage in research [25]. Importantly, we also found that these personal drivers not only motivated patient partners to engage in research, but were also related to what they hoped to achieve from partnering and influenced what they could and were interested in contributing to a study. This indicates that engaging patient partners in early and direct conversations about their motivations for engagement is a good approach towards establishing reciprocal relationships centered around shared values and goals, because it may not only identify what patient partners are hoping to achieve through partnering, but also what they feel they can contribute to the study. Our team’s collective experiences have also taught us that engaging in these upfront conversations helps build trust, mutual respect, and inclusivity and demonstrates the desire to work together to achieve co-created goals. These relational conversations also support moving away from the one-sided (academic researcher-led) onboarding common at the beginning of engagement [26] and help prevent the development of preconceived notions about what patient partners can and should contribute to research. They also help safeguard against tokenism [27], especially if coupled with clearly defined accountability mechanisms that could be co-established through a terms of reference document or the example provided in Additional file 2. Lastly, a better understanding of what motivates patient partners to engage and what they are looking to obtain from the experience (obtained through direct conversations and research) can also be applied to help improve recruitment efforts (by helping to ensure messaging that encompasses potential benefits to engagement that touch upon the different potential motivations for engaging in research), the retention of patient partners in studies (through supporting the creation of desirable experiences that enhance interaction and potential for impact), and ultimately the sustainability and future of patient engagement in research.

In support of other studies which reported diversity in the ways that patient partners are engaged in research [28], our study found that patient partners’ roles and responsibilities vary based on study needs and patient partners’ preferences and capabilities. That said, we also identified different types of contributions that patient partners could make within a study, which were related to patients’ perceptions of the purpose (i.e., illuminate patient and caregiver experience, offer unique perspective, encourage research team to broaden its perspectives beyond academic deliverables, identify approaches and solutions that holistically reflect the entire healthcare ecosystem) and value of patient engagement in research (i.e., desired and actualized personal benefits, personal values, illuminating their own and others’ experiences so as to identify priorities, humanizing the research and ensuring it generates useful information, and ensuring patients have representation at the research table). Taken together, these findings indicate that although patient engagement in research is not a one-size-fits-all approach [16], there are specific considerations that can and should be applied to guide conversations, thinking, and planning about patient partners’ roles and responsibilities and the potential multi-level influences of engagement on the study. Again, engaging in these team-based conversations at the outset of partnership is vital, with the categories of purposes and values identified through this study serving as useful prompts during these conversations–especially with patient partners that are new to the role. Studies have shown that there is a lack of consensus among different stakeholder groups (e.g., patient partners, academic researchers, policymakers) about patient engagement goals, roles, and responsibilities [14, 29–32] and disparities between patient partners expectations for and actual experiences with patient engagement in research [10]. Research on this topic is also very timely due to the increasing focus on establishing the impacts of patient engagement in research [33, 34]. Potential impacts of engagement are varied [16, 34] and multi-level, potentially affecting the individual (i.e., patient partner, academic researcher), research processes, policies and decisions, health outcomes, and social change in health research [35]. Identifying and providing patient partners with the opportunity to realize the full range of roles and responsibilities they can and would like to take on within studies will help support them in maximizing their impact on the study, and thus maximize the impact of engagement on health research.

Although CIHR provides a definition for the term “patient partner”[36], the work of our team and that of others [32, 37] suggests that this term is not universally accepted and may mean different things to different people. This suggests that when initiating partnerships, academic researchers should explain why they use the term and what it means to them, and then discuss what the patients they are partnering with think about the term and whether they have a preferred alternative. Of course, if engaging multiple patient partners in a study, these conversations will likely require some negotiation and compromise to ensure that everyone feels satisfied and represented in the path forward. Further participatory research is needed to explore the suitability of the term “patient partner” in the research setting and identify potential alternatives, as informed by international discussions and the broader participatory action and community-based health research literature [32, 38–40]. Perhaps the term could be replaced by something more general and neutral, such as “patient and public co-researcher”, which could serve as an umbrella term that refers to all individuals with lived/living experience of their or their loved one’s health condition or accessing the healthcare system that are involved in research outside of the role of a study participant. Or, perhaps as suggested by a member of our patient and public advisory (KS) – moving away from language that implies a specific role, to languaging such as “patients and the public engaging in a research study” (PERs) which accommodates the multitudes of ways patients can engage in research. Regardless of whether the term patient partner is replaced, the definition of the term for the role should also clearly include a standardized list of the different terms commonly used to classify patient partners based upon their role within the study and decisions that are made, such as those proposed by IAP2’s spectrum of engagement [22] and built upon by Manafo et al. [23]. As indicated by an interview participant (Katina), such universal standardization of the meaning of the different terms used to refer to patient partners, could for example, then be applied to the development of a matrix to guide conversations between academic researchers and patient partners about their preferred roles and expectations in a project and promote more tangent and consistent information being provided about how patient partners have or will be engaged in a study.

In reflecting upon our study findings, some limitations warrant consideration. First, social desirability bias may have led respondents to answer interviewer questions in a way that cast them in a positive light. We attempted to minimize this bias through careful wording of our questions, introducing the study to and establishing rapport with each participant, and interviewers taking the time to debrief with each other following each interview [41]. Second, the majority of our interview participants were older adult, well-educated, women. All were Caucasian and spoke English fluently. Thus, the perspectives presented in this study may not reflect the experiences of patient partners with more diverse backgrounds, which should be explored in future work. This lack of participant diversity is common in health research [42] and the health system [43] and also likely a reflection of the broader population of patient partners currently engaging in research. It is imperative that future studies actively recruit diverse groups of participants, including applying participatory approaches to identifying and addressing systemic and personal barriers that hinder or prevent participation [44] and as supported by techniques such as maximum variation sampling, if anything is to truly change. Similarly, the fact that study participants were identified through a list of individuals that completed an online survey and interviews were conducted virtually precluded individuals without access to a computer or smartphone and the internet from participating. While this online medium allowed us to interview participants from across Canada, it also likely decreased the likelihood of participation from certain subgroups of the population, such as individuals of low socioeconomic status and from rural and remote communities.

## Conclusions

As patient engagement continues to evolve into a staple of health research, it is critically important that patient partners also have an active say in shaping its future. Otherwise, patient engagement is not truly reflective of the partnership it purports to be and the voices of the ones in power continue to dominate what it is and what it is to become. At the onset of research partnerships, it is the academic researcher’s responsibility to ensure the creation of the time and space necessary to meet with patient partners to discuss and understand each other’s underlying motivations for partnering and the purpose, value, and responsibilities of the patient partner role. These early conversations should help unearth what research partners hope to get out of and feel that they are able to contribute to engaging, and as such contribute to the development of reciprocal relationships that work towards shared and valued goals. Further, since the connotations of the term may vary among individuals, it is also essential that these conversations establish a shared understanding of what is meant and implied by the term patient partner, and that perhaps the term is one day replaced by something that more broadly encompasses the multitudes of lived/living experiences involved in and ways of partnering. Lastly, as the field of patient engagement in research continues to increase its focus on evaluating impacts of engagement, patient partners’ perceptions about the purpose and value of patient engagement in research need to be systematically incorporated to ensure an accurate and complete assessment of the impacts of engagement. It is only through relational and deliberate conversations such as these that patient partners and academic researchers can truly become research partners.

### Supplementary Information

**Additional file 1.** Lay version of the manuscript.

**Additional file 2.** Overview.

**Additional file 3**. GRIPP2 short form checklist.

**Additional file 4.** Semi-structured interview guide.

## Data Availability

The datasets used and/or analyzed during the current study are available from the corresponding author on reasonable request.

## Abbreviations

CIHR: Canadian Institutes of Health Research
IAP2: International Association for Public Participation
PERs: Patients and the public engaging in a research study
SPOR: Strategy for Patient-Oriented Research

## References

[1] Canadian institutes of health research. Health research in Canada and you [Available from: https://cihr-irsc.gc.ca/e/43753.html#Health.

[2] Canadian institutes of health research. Strategy for POR - patient engagement framework 2014 [Available from: http://www.cihr-irsc.gc.ca/e/48413.html.

[3] Tebes JK, Thai ND (2018). Interdisciplinary team science and the public: steps toward a participatory team science. Am Psychol.

[4] Salas E, Shuffler ML, Thayer AL, Bedwell WL, Lazzara EH (2015). Understanding and improving teamwork in organizations: a scientifically based practical guide. Hum Resour Manage.

[5] Wallerstein N, Calhoun K, Eder M, Kaplow J, Wilkins CH (2019). Engaging the community: community-based participatory research and team science.

[6] Carroll SL, Embuldeniya G, Abelson J, McGillion M, Berkesse A, Healey JS (2017). Questioning patient engagement: research scientists’ perceptions of the challenges of patient engagement in a cardiovascular research network. Patient Prefer Adherence.

[7] Elliott MJ, McCarron TL, Schick-Makaroff K, Getchell L, Manns B, Fernandez N (2023). The dynamic nature of patient engagement within a Canadian patient-oriented kidney health research network: perspectives of researchers and patient partners. Health Expect.

[8] Clark PR (2009). Teamwork: building healthier workplaces and providing safer patient care. Crit Care Nurs Q.

[9] Hemphill R, Forsythe LP, Heckert AL, Amolegbe A, Maurer M, Carman KL (2020). What motivates patients and caregivers to engage in health research and how engagement affects their lives: qualitative survey findings. Health Expect.

[10] Jordan M, Rowley E, Morriss R, Manning N (2015). An analysis of the research team-service user relationship from the service user perspective: a consideration of ‘the three Rs’ (roles, relations, and responsibilities) for healthcare research organisations. Health Expect.

[11] Madison S, Colon-Moya AD, Morales-Cosme W, Lorenzi M, Diaz A, Hickson B (2022). Evolution of a research team: the patient partner perspective. Res Involve Engagem.

[12] Musson LS, McDermott CJ, Hobson EV (2019). Exploring patient and public involvement in motor neuron disease research. Amyotroph Lateral Scler Frontotemporal Degener.

[13] Schilling I, Behrens H, Hugenschmidt C, Liedtke J, Schmiemann G, Gerhardus A (2019). Patient involvement in clinical trials: motivation and expectations differ between patients and researchers involved in a trial on urinary tract infections. Res Involve Engag.

[14] Thompson J, Bissell P, Cooper CL, Armitage CJ, Barber R (2014). Exploring the impact of patient and public involvement in a cancer research setting. Qual Health Res.

[15] Ashcroft J, Wykes T, Taylor J, Crowther A, Szmukler G (2016). Impact on the individual: what do patients and carers gain, lose and expect from being involved in research?. J Ment Health.

[16] Chudyk AM, Stoddard R, McCleary N, Duhamel TA, Shimmin C, Hickes S (2022). Activities and impacts of patient engagement in CIHR SPOR funded research: a cross-sectional survey of academic researcher and patient partner experiences. Res Involve Engag.

[17] Thorne S (2008). Interpretive description.

[18] Crotty MJ. The foundations of social research: Meaning and perspective in the research process. In: The foundations of social research, vol 1, p. 256 1998.

[19] Lincoln YS, Lynham SA, Guba EG (2011). Paradigmatic controversies, contradictions, and emerging confluences, revisited. Sage Handb Qual Res.

[20] Staniszewska S, Brett J, Simera I, Seers K, Mockford C, Goodlad S, et al. GRIPP2 reporting checklists: tools to improve reporting of patient and public involvement in research. bmj. 2017;358. 10.1136/bmj.j3453 PMC5539518 28768629

[21] Braun V, Clarke V (2022). Conceptual and design thinking for thematic analysis. Qualitative Psychol.

[22] International association for public participation. IAP2's public participation spectrum [Available from: https://www.iap2.org.au/Tenant/C0000004/00000001/files/IAP2_Public_Participation_Spectrum.pdf.

[23] Manafò E, Petermann L, Vandall-Walker V, Mason-Lai P (2018). Patient and public engagement in priority setting: a systematic rapid review of the literature. PLoS ONE.

[24] Braun V, Clarke V (2023). Toward good practice in thematic analysis: Avoiding common problems and be (com) ing a knowing researcher. Int J Transgender Health.

[25] Tarpey M. Why people get involved in health and social care research: Involve Eastleigh; 2006. https://www.invo.org.uk/wp-content/uploads/documents/whypeoplegetinvolvedinresearch2006.pdf.

[26] Dudley L, Gamble C, Allam A, Bell P, Buck D, Goodare H (2015). A little more conversation please? Qualitative study of researchers’ and patients’ interview accounts of training for patient and public involvement in clinical trials. Trials.

[27] Hahn DL, Hoffmann AE, Felzien M, LeMaster JW, Xu J, Fagnan LJ (2017). Tokenism in patient engagement. Fam Pract.

[28] Bird M, Ouellette C, Whitmore C, Li L, Nair K, McGillion MH (2020). Preparing for patient partnership: a scoping review of patient partner engagement and evaluation in research. Health Expect.

[29] Bellows M, Kovacs Burns K, Jackson K, Surgeoner B, Gallivan J (2015). Meaningful and effective patient engagement: what matters most to stakeholders. Patient Exp J.

[30] Braun V, Clarke V (2006). Using thematic analysis in psychology. Qual Res Psychol.

[31] Boudes M, Robinson P, Bertelsen N, Brooke N, Hoos A, Boutin M (2018). What do stakeholders expect from patient engagement: are these expectations being met?. Health Expect.

[32] Gallivan J, Kovacs Burns K, Bellows M, Eigenseher C (2012). The many faces of patient engagement. J Particip Med.

[33] L’Espérance A, O’Brien N, Grégoire A, Abelson J, Canfield C, Del Grande C (2021). Developing a Canadian evaluation framework for patient and public engagement in research: study protocol. Res Involv Engag.

[34] Modigh A, Sampaio F, Moberg L, Fredriksson M (2021). The impact of patient and public involvement in health research versus healthcare: a scoping review of reviews. Health Policy.

[35] Aubin D, Hebert M, Eurich D (2019). The importance of measuring the impact of patient-oriented research. CMAJ.

[36] Canadian institutes of health research. Ethics guidance for developing partnerships with patients and researchers. 2020.

[37] Rolfe DE, Ramsden VR, Banner D, Graham ID (2018). Using qualitative health research methods to improve patient and public involvement and engagement in research. Res Involv Engag.

[38] Tritter JQ (2009). Revolution or evolution: the challenges of conceptualizing patient and public involvement in a consumerist world. Health Expect.

[39] Frank L, Morton SC, Guise J-M, Jull J, Concannon TW, Tugwell P (2020). Engaging patients and other non-researchers in health research: defining research engagement. J Gen Intern Med.

[40] Sanders Thompson VL, Ackermann N, Bauer KL, Bowen DJ, Goodman MS (2021). Strategies of community engagement in research: definitions and classifications. Translational Behav Med.

[41] Bergen N, Labonté R (2020). “Everything is perfect, and we have no problems”: detecting and limiting social desirability bias in qualitative research. Qual Health Res.

[42] Routen A, Bodicoat D, Willis A, Treweek S, Paget S, Khunti K (2022). Tackling the lack of diversity in health research. Br J Gen Pract.

[43] Abelson J, Canfield C, Leslie M, Levasseur MA, Rowland P, Tripp L (2022). Understanding patient partnership in health systems: lessons from the Canadian patient partner survey. BMJ Open.

[44] Manalili K, Siad FM, Antonio M, Lashewicz B, Santana MJ (2022). Codesigning person-centred quality indicators with diverse communities: a qualitative patient engagement study. Health Expect.

