# Publisher Correction to: Exploring patient and caregiver perceptions of the meaning of the patient partner role: a qualitative study

**DOI:** 10.1186/s40900-024-00571-5

**Published:** 2024-04-26

**Authors:** Anna Maria Chudyk, Roger Stoddard, Nicola McCleary, Todd A. Duhamel, Carolyn Shimmin, Serena Hickes, Pan-Canadian group of patient and public advisors, Annette S. H. Schultz

**Affiliations:** 1 Department of Family Medicine, Rady Faculty of Health Sciences, University of Manitoba, CR3024 ‑ 369 Tache Avenue, R2H 2A6 Winnipeg, MB Canada; 2 Horizon Health Network, 80 Woodbridge Street, E3B 4R3 Fredericton, NB Canada; 3 Ottawa Hospital Research Institute - Clinical Epidemiology ProgramK1H 8L6, Room L1202, Box 711 ‑ 501 Smyth Road, Ottawa, ON Canada; 4 School of Epidemiology and Public Health, University of Ottawa, 600 Peter Morand Crescent, K1G 5Z3 Ottawa, ON Canada; 5 Faculty of Kinesiology and Recreation Management, 212 Active Living Centre, R3T 2N2 Winnipeg, MB Canada; 6 6Institute of Cardiovascular Sciences, St. Boniface General Hospital Albrechtsen Research Centre, R4012 ‑ 351 Tache Ave, R2H 2A6 Winnipeg, MB Canada; 7 George and Fay Yee Centre for Healthcare Innovation, 3rd floor– 753 McDermot Avenue, R3E 0T6 Winnipeg, MB Canada; 8 Translating Emergency Knowledge for Kids (TREKK) Parent Advisory Group, Children’s Hospital Research Institute of Manitoba, 512E ‑ 715 McDermot Avenue, R3E 3P4 Winnipeg, MB Canada; 9 College of Nursing, Rady Faculty of Health Sciences, University of Manitoba, CR3022, 369 Tache Avenue, R2H 2A6 Winnipeg, MB Canada

**Publisher Correction to**: ***Res Involv Engagem*9, 106 (2023).**

10.1186/s40900-023-00511-9

Following publication of the original article [1], the authors identified an error in the order of the authors since Annette S. H. Schultz should be indicated as the last author instead of the Pan-Canadian group of patient and public advisors. The publisher apologises to the authors and readers for the inconvenience caused by this mistake.

The incorrect author list reads:

Anna Maria Chudyk1*, Roger Stoddard2, Nicola McCleary3,4, Todd A. Duhamel5,6, Carolyn Shimmin7,

Serena Hickes8, Annette S. H. Schultz9 and Pan-Canadian group of patient and public advisors

The correct author list should read:

Anna Maria Chudyk1*, Roger Stoddard2, Nicola McCleary3,4, Todd A. Duhamel5,6, Carolyn Shimmin7,

Serena Hickes8, Pan-Canadian group of patient and public advisors and Annette S. H. Schultz9

All the authorship changes are implemented in this correction and the original article [1] has been corrected.
