# Supplementary material for: Exploring patient and caregiver perceptions of the meaning of the patient partner role: a qualitative study
Source: Res Involv Engagem. 2023 Nov 28;9:106. doi: 10.1186/s40900-023-00511-9 (PMC10683322; doi:10.1186/s40900-023-00511-9)
Supplement: Supplementary file 1 — Additional file 1. Lay version of the manuscript. [file 40900_2023_511_MOESM1_ESM.pdf]

Lay Summary

Exploring patient and caregiver perceptions of the meaning of the patient partner role: A qualitative study

Document overview

This document provides a high-level overview ("lay summary") of our publicly available journal article:

Exploring patient and caregiver perceptions of the meaning of the patient partner role: A qualitative study. Published in Research Involvement and Engagement. Authored by: Anna Maria Chudyk, Roger Stoddard, Nicola McCleary, Todd A. Duhamel, Carolyn Shimmin, Serena Hickes, Patient and Public Advisors, Annette SH Schultz.

It was created with the intention of sharing our study's findings and implications to a wider audience. To help meet this intention, we:

- PAGE 1: Briefly summarize our study's key background, design, and analysis information on this first page;

- PAGES 2-7: Highlight our study's key findings (and their implications) according to the four main questions that were used to explore participants' motivations for becoming patient partners and their understanding of the role.

Study background

Patients and their unpaid caregivers (e.g., family and friends) have direct lived/living experience of health, illness, and accessing health care services. Patient engagement in research applies this lived/living experience to shaping why and how research is carried out by including patients and caregivers as members of the research team. Patients and caregivers that take on this researcher role are referred to as patient partners.

Since patients and caregivers are more commonly involved in research as study participants, patient partners and academic researchers (scientists) have expressed uncertainty about how to best work together as research partners. A key characteristic of successful partnerships is a shared understanding of, and respect for, each others' motivations and expectations, and the unique knowledge and skillsets they contribute to reaching the team's goals. There is a lack of studies that have explored these motivations and role expectations in Canadian patient partners.

Therefore, **this study aimed to explore Canadian patient partners' motivations for engaging in research and understanding of their role.**

Methods

Study design: Interviews in which participants were asked pre-planned questions were co-conducted by a patient partner (RS) and academic researcher (AMC).

Participants: 13 individuals with prior experience of being patient partners on projects funded through the Strategy for Patient-Oriented Research. These individuals self-identified as being interested in taking part in the interviews when they participated in our previous study [Chudyk AM et al. Res Inv Eng 2022 Dec; 8(1):1-4]. To be included as study participants, they had to be able to communicate in English and give informed consent to participate.

Data collection: Interviews took place February - April 2021 over Zoom. They lasted 60-90 minutes, followed an interview guide developed by our research team, and were recorded and transcribed.

Data analysis: Thematic analysis -- a method that involves looking for common patterns (themes) among participants' answers to questions.

Patient engagement: A patient partner (RS) co-designed and co-led this study. Another patient partner (SH) shared ideas and feedback at major study milestones. Study participants were consulted on the accuracy and interpretation of the study findings and invited to co-author the journal artilce (as part of the Patient and Public Advisors group).

A What are patient partners' reasons for becoming a patient partner?

Participants' experiences of a health condition and/or accessing healthcare services provided them with the lived experience needed to be a patient partner.

They also had unique personal reasons for wanting to engage in research that also influenced what they hoped to achieve from partnering. These included:

- **Altruism -** for example, the desire to help others in similar situations, to give back, devote yourself to tasks that are greater than you.
- **Professional background -** influenced what patient partners could contribute to the research team (beyond their lived experience), their general research interests, their belief in their ability to be a patient partner, and their view of research as a natural extension of their professional role.
- **Desire for personal growth and expression -** for example, the opportunity to be challenged mentally and learn new things, be creative, expand social and professional networks, and feel good about yourself.
- **Personal history -** for example, prior research involvement, sharing or seeing family members share their healthcare experiences with others.

Why it matters
(in terms of contributing to the development of more meaningful relationships):

Taking the time to learn about and discuss patient partners' reasons for becoming a patient partner can help academic researchers get a better idea of the wide range of skills and experiences that patient partners bring to the table, as well as what they are hoping to get out of partnering. This information can also:

- help research partners develop a reciprocal relationship in which the needs and wants of all parties are better understood and met;

- positively influence academic researchers' decisions to engage patient partners in the future;

- help recruit and retain patient partners (by better understanding what motivates them and what they are looking for from the experience).

B How do patient partners define the term "patient partner"?

Key Terms

"Patient"

A person that brings the viewpoint and experiences of a client of the healthcare system, and that can speak to their own or their loved one's health and/or medical experiences.

- ***Key consideration:*** Is being a patient specific to a point in time or situation (context)? For example, are you still a patient when "cured" or not at the doctor's office?

"Partner"

A person with viewpoints and experiences that are heard by the other members of the research team and meaningfully affect the research.

- ***Key consideration:*** If being a partner is interpreted as meaning a person has an equal say in a relationship, including in the decisions that are made, then is the term 'patient partner' an appropriate catch-all term for the role?

Why this matters

Understanding how patient partners define the term gives insights into the experiences they feel they bring to the table and expectations they may have about the power dynamics in the role (e.g., equality, influence on decision-making). It can also influence whether someone considers themselves to be eligible for the role (e.g., someone may think they can only be a patient partner if/when they are "sick").

C What do patient partners see as the purpose of being a patient partner?

Study-specific puposes

Give voice to the patient/caregiver experience

Ideally, this perspective is what drives the research -- which can only happen if the research team values lived/living experience.

Offer a unique perspective

That may provide practical ideas and answers to strategizing research problems, push academic researchers to consider and explain what the research is going to lead to and how it will ultimately affect patient lives, give the study more credibility, and enhance patient, caregiver, and patient partner experiences with research.

Systems-level puposes

Encourage the research team to broaden its focus

- ***beyond the production of academic deliverables*** to the application of research findings to enhancing patient and caregiver experiences within the healthcare system and patient outcomes.
- to **explore avenues through which the study can impact the system** and the system's impact on patient outcomes.

Work in synergy with the interdisciplinary research team to identify approaches and solutions that holistically reflect the realities, experiences, and possibilities of the entire healthcare ecosystem.

D What do patient partners see as the value of patient partners?

Participants' discussion of the value of patient partners focused on the personal values that they got from and brought to the role, as well as the overall value of patient partners to health research.

Personal values

- The ***desired/actual personal benefits*** obtained from partnering. They are oftentimes related to their reasons for becoming patient partners and may change over time.
- ***Principles*** brought to the role (e.g., respect, mutual learning, authenticity) that shape patient partners' expectations for how they and others should be treated and the nature of the engagement activities.

Value to health research

Giving voice to their personal and networks' experiences of being a patient, caregiver, and/or the healthcare system. This sharing of experience is important because it:

- ***gives others an idea of what is important*** to a person, or the caregiver of a person, living with a condition or accessing healthcare services and may in turn affect research directions and outcomes in many ways (e.g., expanding the focus of the research question, bringing forward novel ideas);
- ***helps "humanize" the research*** through personal stories and contributing practical perspectives to scientific points of view;
- ***helps ensure that the research generates useful information*** (to a person, or a caregiver of a person, living with the condition) by, for example, suggesting important outcomes to use;
- ensures that there is someone at the research table whose ***primary focus is representing the patient/caregiver experience.***

E

What do patient partners see as their roles and/or responsibilities?

Three key roles

1. Ambassador for the patient/caregiver perspective

- Represent the patient/caregiver perspective at the research table
  - some patient partners also take this to also mean drawing on networks to gather feedback and perspectives of the wider patient and caregiver community
- Ensure that the voices of other patient partners, patients, and caregivers are being respected and informing research activities

2. Contribute to the research process:

- Actual roles and responsibilities are fluid (vary by project and patient partner) and are decided upon through discussions between researcher partners (preferable) or largely dictated by the academic researcher members of the team (unfavorable but oftentimes a reality).
- How patient partners contribute to the research process should be tailored to meet the study's needs and goals, and patient partner preferences and capabilities.
- Examples of patient partner roles and responsibilities in contributing to the research process include...

3. Two-way communication

- (see next page)

Examples of roles in the research process:

- consultant, advisor, advisory panel co-lead, co-investigator, co-author.

Examples of responsibilities

Study-level:

- participate in team meetings,
- contribute real-world ideas and perspectives,
- help define and answer research questions,
- help identify priorities, themes, and outcomes important to patients,
- improving the accessibility of the study's language,
- help with study planning,
- help increase the number and diversity of participants recruited into the study,
- help make sense of findings and put them into context,
- help design study outputs (e.g., journal articles, reports) and share and present them.

Person-level:

- honor your commitment to the task,
- provide what you can, when you can, while acknowledging that sometimes your or your loved one's personal circumstances may prevent you from being able to engage at a given time,
- be respectful of everyone at the table,
- be flexible when giving input,
- maintain the integrity of the research,
- help ensure that other patients and caregivers have a voice in research,
- advance the concept of patient engagement in research.

E

What do patient partners see as their roles and/or responsibilities?

Three key roles *(continued)*

3. Two-way communication

Cross-cutting theme

- This theme spanned both 'ambassador for the patient and caregiver voice' and 'contribute to the research process' themes

Sharing information

- Included sharing healthcare experiences openly with the research team and others; providing honest, objective, and constructive feedback to the research team about the research, general engagement process, and other factors relevant to the partnership; and open communication (including about needs).
    - May be best supported through the presence of a designated point-person (like a patient engagement facilitator).

Receiving information

- Included listening; being open to and respectful of others' perspectives; realizing that other viewpoints are also present at the research table; engaging in mutually respectful discussions that help to build a more dynamic research team; breaking down walls between patient partners and the other members of the research team; and raising the profile of patient partners through true partnership with academic researchers.
    - Doing so opens the space for patient partner perspectives to interact synchronistically with the other perspectives at the research table to enhance research directions and processes in a way that wouldn't be possible if any of the partners at the table were missing.

Acknowledgments

Our research team extends their most sincere gratitude to:

- all of the interview participants for sharing their experiences, stories, and perspectives and for being so engaged throughout the study;
- Ms. Sandra Dalziel, Ms. Delane Linkiewich, Ms. Lesley Singer, Mr. Kurt Schreiner, Ms. Kathy Smith, and Ms. Janice Sumpton for extending their study participation to include authorship as part of the Patient and Public Advisors;
- Ms. Annette Greenwood for helping develop the study's Supplementary Material #2;
- Mr. Patrick Faucher for being an invaluable part of our research endeavor through his creation of key participant facing materials and the paper's tables and figures, as well as the George & Fay Yee Centre for Healthcare Innovation for Mr. Faucher's services;
- Mr. Levi Klassen for his assistance in informing this study's literature review and supporting the study as a summer practicum student
- Ms. Sasha Kullman for assistance with manuscript revisions;
- the College of Nursing (University of Manitoba) for helping supporting this paper's open access publication fees;
- the University Collaborative Research Program (University of Manitoba, grant number 50664) for funding the study;
- the Canadian Institutes of Health Research for supporting Dr. Chudyk's postdoctoral fellowship through a Patient-Oriented Research Awards-Transition to Leadership Stream award (grant number 170670).
