# Supplementary material for: Exploring patient and caregiver perceptions of the meaning of the patient partner role: a qualitative study
Source: Res Involv Engagem. 2023 Nov 28;9:106. doi: 10.1186/s40900-023-00511-9 (PMC10683322; doi:10.1186/s40900-023-00511-9)
Supplement: Supplementary file 2 — Additional file 2. Overview. [file 40900_2023_511_MOESM2_ESM.docx]

**Overview**

This document was created as a Supplementary Material to our study:

Exploring patient and caregiver perceptions of the meaning of the patient partner role: A qualitative study. Published in Research Involvement and Engagement. Authored by: Anna Maria Chudyk, Roger Stoddard, Nicola McCleary, Todd A. Duhamel, Carolyn Shimmin, Serena Hickes, Patient and Public Advisors, Annette SH Schultz.

**Definitions of key terms**

**Patient**: A person that brings the viewpoints and experiences of a client of the healthcare system, and that can speak to their own or their loved one’s health and/or medical experiences.

**Patient partner:** A patient with viewpoints and experiences that are heard by the other members of the research team and meaningfully affect the research. This person is meaningfully and actively engaged in the research beyond the role of a study participant.

**Academic researcher:** A person that has received formal (usually graduate school level) training in the design and conduct of health research and is responsible for applying this training to guide or conduct the research team’s activities.

**What this document sets out to accomplish:**

Our study aimed to explore patient partners’ motivations for engaging in research and their understanding of the purpose, value, and responsibilities of the patient partner role. We found that engaging in these discussions can help identify what patient partners hope to get out of, and feel that they are able to contribute to, their role. We also found that patient partners assign different meanings to the term patient partner, which can in turn impact what they expect from the role. Based on these findings, we recommended that academic researchers and patient partners engage in discussions about their motivations and expectations for engaging in research at the outset of their partnership. These discussions are an important first step towards the development of reciprocal relationships built upon a mutual understanding of, and desire to work towards, shared and valued goals.

Consequently, we developed this document to guide academic researchers and patient partners in developing a shared understanding of each others’ motivations and expectations for engaging in research together. Specifically, this document consists of three conceptually similar versions of the same form:

- Patient partner version (pages 4-11)
- Academic researcher version (pages 12-19)
- Consolidated version (pages 20-27)

It is important to engage patient partners early (like when research ideas are being generated and research questions defined). Therefore, this document will ideally be applied to help plan engagement activities when a study is being developed. That said, this document can also be used when a patient partner newly joins a study’s ongoing engagement activities, to plan new engagement activities within an ongoing study, or to engage in these conversations for the first time when a study is well underway (as the saying goes – better late than never).

**Instructions**

The individuals responsible for initiating their study’s engagement activity are instructed to go through all three versions of the form and tailor the [orange text] to reflect the details of their study.

Next, the patient partner(s) and academic researcher(s) that will be working most closely together on the study are to each fill out their respective version of the form.

After respondents have had the time to think and reflect on their own, they should all come together to discuss their answers and jointly complete the consolidated version of the form, which reflects their shared understanding of the main takeaways from their discussions.

This activity is meant as a starting point for negotiating a shared understanding of the role of patient partners within a study. It leads nicely into, for example, the co-development of a terms of reference that formally outlines a study’s engagement activities.

**Supplementary resources:**

In working through this form, you might find it helpful to refer to the following *outside resources* to help inform your thinking about the following aspects of engagement:

**Guiding framework of engagement:** Canadian Institutes of Health Research.

Strategy for Patient Oriented Research - Patient Engagement Framework. Available at: <http://www.cihr-irsc.gc.ca/e/48413.html> .

**Levels of engagement:**

International Association for Public Participation. IAP2's Public Participation Spectrum. Available at: <https://www.iap2.org.au/Tenant/C0000004/00000001/files/IAP2_Public_Participation_Spectrum.pdf>.

Manafò E, Petermann L, Vandall-Walker V, Mason-Lai P. [Patient and public engagement in priority setting: a systematic rapid review of the literature](https://journals.plos.org/plosone/article?id=10.1371/journal.pone.0193579). PloS one. 2018;13(3):e0193579.

You may also want to refer to the following *free resources from our research group* to help inform your thinking about the following aspect of patient engagement:

**Models and frameworks of patient engagement:** Chudyk AM, Horrill T, Waldman C, Demczuk L, Shimmin C, Stoddard R, Hickes S, Schultz AS. Scoping review of models and frameworks of patient engagement in health services research. BMJ open. 2022 Aug 1;12(8):e063507.

**Developing a terms of reference:** Multimedia Appendix 1 in Chudyk AM, Ragheb S, Kent D, Duhamel TA, Hyra C, Dave MG, Arora RC, Schultz AS. Patient engagement in the design of a mobile health app that supports enhanced recovery protocols for cardiac surgery: development study. JMIR Perioperative Medicine. 2021 Nov 30;4(2):e26597.

**Engaging patient partners:** Chudyk AM, Stoddard R, McCleary N, Duhamel TA, Shimmin C, Hickes S, Schultz AS. Activities and impacts of patient engagement in CIHR SPOR funded research: a cross-sectional survey of academic researcher and patient partner experiences. Research Involvement and Engagement. 2022 Dec;8(1):1-4.

1. **Overview**

This form intends to help patient partner(s) and academic researcher(s) explore each others’ motivations and expectations for their research partnership. In doing so, this form will help them identify matching and/or differing perspectives, expectations, or points of view that will help them better understand each other and establish a reciprocal relationship based on mutual understanding and common goals. The patient partner(s) and academic researcher(s) that will be working most closely together on the study will fill out similar versions of this form and then meet to discuss and explore their answers as a step towards jointly negotiating the terms of their partnership.

***1.1 Instructions***

Only answer the questions you are comfortable with. Let [state point person’s name and preferred contact information] know if you are unsure of anything!

1. **Key terms**

***2.1 Patient partner***

The Canadian Institutes of Health Research is one of the key funders of Canadian research. Based on their suggested terminology, we have chosen to use the term **patient partner** to refer to your position within our research team. We define this term as *[definition]*.

Although we use this term throughout this form, we’d like to get your feedback on whether…

1. You are ok with us using this term to refer to your position:

Yes  No  Unsure

1. What the term patient partner implies to you, and whether there is a term you’d prefer we use instead of patient partner (including any reasons you’d like to share about why you prefer this term):

***2.2 [Proposed level of engagement]***

When thinking about different ways that patient partners can be engaged in research studies, researchers sometimes start by considering: (a) the influence that patient partners will have on the decisions that are made within the study and (b) whether patient partners are engaged throughout the study or within a certain stage of the study. The answers to these questions guide the “level” at which patient partners will be engaged.

We are open to engaging you [at a single stage/across multiple stages – please define which ones using simple language and the anticipated time frame/date range for each stage] of our study and to have your ideas and input [be taken into consideration when/be weighed equally when/determine what] decisions are made about the study. Because of this, we initially propose for you to be engaged at the level(s) of [consult/involve/collaborate/lead 🡪 choose any/all that apply].

1. Please let us know what your thoughts are on your potential level(s) of engagement in this study, including any preferences that you have:

1. **Getting to know you**

***3.1 How you want to be addressed***

1. What is your preferred name (the name that you’d like us to call you):
2. What pronouns do you go by (e.g., they, she/her, he/him):
3. What salutations would you like us to use when referring to you (e.g., Dr., Mx., Mr., Ms.):
   1. ***Why you became a patient partner***
4. What are your reasons for wanting to be a patient partner on this study:

1. Please describe any life experiences that might shape how and what you can contribute to this study:

***Some potential examples include:*** *Experiences with your or a loved one’s health conditions and/or accessing healthcare services; skills or knowledge acquired through volunteering, hobbies, work, and/or school; previous experience with research and/or engagement; etc.*

- 1. ***Initial ideas about your potential roles/responsibilities within this study***

Here is a brief summary of our study that may help you answer the questions that come next *[below are some possible key points to include in the summary]*

Our study aims to *[please use straightforward language and complete ahead of sharing the form with a patient partner – for example, “… determine whether adult women’s physical activity levels are associated with mental well-being.]*

We propose to meet this aim through *[provide a straightforward description of your study design, including a high-level overview of what data will be collected and how – for example, “… a cross-sectional study, meaning that we will collect participant data at a single point in time. We will measure participants’ physical activity by asking them to wear a device called an accelerometer for a week, and mental well-being and background (sociodemographic) characteristics through questionnaires that they will fill out at a single data collection session at our research center.]*

We have already/we are in the stages of *[what work has already been done of the study – for example, “We have already received grant funding to support this study.”]*.

During our research partnership, the study will be going through the following stages of the research cycle *[list stage(s) and provide straightforward example(s) of what the stage(s) involve(s) if this is potentially unclear (using bullet points will make the information easiest to follow across many stages) – for example: finalizing our study plan; recruitment – identifying and approaching individuals to participate in the study (based on our established protocol); data collection; data analysis; data synthesis (making sense of our findings; knowledge translation (formally writing up our findings and sharing them through journal publications, reports, and presentations)]*

- 1. ***How you view the patient partner role***

1. What do you see as the purpose and/or value in being a patient partner (in general and/or specific to this study):

1. What do you see as the roles and/or responsibilities of a **patient partner** (in general and/or specific to this study)? These could, for example, refer to how you contribute to team dynamics or the research activities:

1. What do you see as **academic researchers’** roles and/or responsibilities when engaging patient partners:

1. Based on the brief description of our study, are there any roles or responsibilities that interest you in terms of how you could be engaged in this study? Are there any potential barriers (that you’d like to share) that could prevent you from engaging, or supports that you may require to fully engage, in these roles and responsibilities? Please remember that this is just a starting point that we will build on as we co-develop another document (terms of reference) to continue to guide our work together:

1. **Overview**

This form intends to help patient partner(s) and academic researcher(s) explore each others’ motivations and expectations for their research partnership. In doing so, this form will help them identify matching and/or differing perspectives, expectations, or points of view that will help them better understand each other and establish a reciprocal relationship based on mutual understanding and common goals. The patient partner(s) and academic researcher(s) that will be working most closely together on the study will fill out similar versions of this form and then meet to discuss and explore their answers as a step towards jointly negotiating the terms of their partnership.

***1.1 Instructions***

Only answer the questions you are comfortable with. Let [state point person’s name and preferred contact information] know if you are unsure of anything!

1. **Key terms**

***2.1 Patient partner***

The Canadian Institutes of Health Research is one of the key funders of Canadian research. Based on their suggested terminology, we have chosen to use the term **patient partner** to refer to our patient co-researchers. We define this term as *[definition]*.

1. Although we use this term throughout this form, we are also asking patient partners’ preferences surrounding its use.
2. Please share what the term patient partner implies to you and any other perspectives that you have on this term that you would like to contribute to the conversation about its use:

***2.2 [Proposed level of engagement]***

When thinking about different ways that patient partners can be engaged in research studies, researchers sometimes start by considering: (a) the influence that patient partners will have on the decisions that are made within the study and (b) whether patient partners are engaged throughout the study or within a certain stage of the study. The answers to these questions guide the “level” at which patient partners will be engaged.

We are open to engaging patient partners [at a single stage/across multiple stages] of our study and to have their ideas and input [be taken into consideration when/be weighed equally when/determine what] decisions are made about the study. Because of this, we initially propose for patient partners to be engaged at the level(s) of [consult/involve/collaborate/lead 🡪 choose any/all that apply].

1. Please let us know what your thoughts are on patient partners’ potential level(s) of engagement in the study, including any preferences you have:

1. **Getting to know you**

***3.1 How you want to be addressed***

1. What is your preferred name (that you’d like us to call you):
2. What pronouns do you go by (e.g., they, she/her, he/him):
3. What salutations would you like us to use when referring to you (e.g., Dr., Mx., Mr., Ms.):
   1. ***Motivations for engagement***
4. What are your reasons for wanting to engage patient partner(s) on this study:

1. Please describe any life experiences that shape how and what you can contribute to this study:

***Some potential examples include:*** *Experiences with your or a loved one’s health conditions and/or accessing healthcare services; skills or knowledge acquired through volunteering, hobbies, work, and/or school; previous experience with research and/or engagement; etc.*

- 1. ***Initial ideas about patient partners’ potential roles/responsibilities within this study***

Here is a brief summary of our study that may help you answer the questions that come next *[below are some possible key points to include in the summary]*

Our study aims to *[please use straightforward language and complete ahead of sharing the form with a patient partner – for example, “… determine whether adult women’s physical activity levels are associated with mental well-being.]*

We propose to meet this aim through *[provide a straightforward description of your study design, including a high-level overview of what data will be collected and how – for example, “… a cross-sectional study, meaning that we will collect participant data at a single point in time. We will measure participants’ physical activity by asking them to wear a device called an accelerometer for a week, and mental well-being and background (sociodemographic) characteristics through questionnaires that they will fill out at a single data collection session at our research center.]*

We have already/we are in the stages of *[what work has already been done of the study – for example, “We have already received grant funding to support this study.”]*.

During our research partnership, the study will be going through the following stages of the research cycle *[list stage(s) and provide straightforward example(s) of what the stage(s) involve(s) if this is potentially unclear (using bullet points will make the information easiest to follow across many stages) – for example: finalizing our study plan; recruitment – identifying and approaching individuals to participate in the study (based on our established protocol); data collection; data analysis; data synthesis (making sense of our findings; knowledge translation (formally writing up our findings and sharing them through journal publications, reports, and presentations)]*

- 1. ***How you view the patient partner role***

1. What do you see as the purpose and/or value of patient partners (in general and/or specific to this study):

1. What do you see as the general roles and/or responsibilities of patient partners (in general and/or specific to this study)? These could, for example, refer to your responsibilities to the research team dynamics or the research activities:

1. What do you see as **academic researchers’** roles and/or responsibilities when engaging patient partners:

1. Based on the brief description of our study, are there any roles or responsibilities that jump out at you in terms of how patient partners could be engaged in this study? Can you think of any potential barriers that could prevent them from engaging, or supports that they may require to fully engage in these roles and responsibilities? Please remember that this is just a starting point that we will build on as we co-develop another document (terms of reference) to continue to guide our work together:

1. **Overview**

This form intends to help patient partner(s) and academic researcher(s) explore each others’ motivations and expectations for their research partnership. In doing so, this form will help them identify matching and/or differing perspectives, expectations, or points of view that will help them better understand each other and establish a reciprocal relationship based on mutual understanding and common goals. The patient partner(s) and academic researcher(s) that will be working most closely together on the study will fill out similar versions of this form and then meet to discuss and explore their answers as a step towards jointly negotiating the terms of their partnership.

***1.1 Instructions***

Please document your shared understanding of the main-takeaways that arose from discussing research partners’ individual answers to this form.

1. **Key terms**

***2.1 Patient partner***

The Canadian Institutes of Health Research is one of the key funders of Canadian research. Based on their suggested terminology, we have chosen to use the term **patient partner** to refer to our patient co-researchers. We define this term as [definition].

1. Have you jointly agreed to use this term in this study:

Yes  No  Unsure – needs further discussion

1. What are the main takeaways from your discussions about this term (including any alternate terminology you’ve agreed to use):

***2.2 [Proposed level of engagement]***

When thinking about different ways that patient partners can be engaged in research studies, researchers sometimes start by considering: (a) the influence that patient partners will have on the decisions that are made within the study and (b) whether patient partners are engaged throughout the study or within a certain stage of the study. The answers to these questions guide the “level” at which patient partners will be engaged.

We are open to engaging you [at a single stage/across multiple stages – please define which ones using simple language and the anticipated time frame/date range for each stage] of our study and to have your ideas and input [be taken into consideration when/be weighed equally when/determine what] decisions are made about the study. Because of this, we initially propose for you to be engaged at the level(s) of [consult/involve/collaborate/lead 🡪 choose any/all that apply].

1. What are the main takeaways about patient partners’ potential level(s) of engagement in the study, including any preferences that patient partner(s) and/or academic researcher(s) have for the level of engagement:

1. **Getting to know everyone**

***3.1 Please list the first names, pronouns, and salutations that the group that completed this activity would like to go by:***

- 1. ***Motivations for engaging patient partners***

1. What are the motivations/reasons for engagement in this study:

1. What are the different life experiences that shape how and what the group that completed this form can contribute to the study:

***Some potential examples include:*** *Experiences with your own or a loved one’s health conditions and/or accessing healthcare services; skills or knowledge acquired through volunteering, hobbies, work, and/or school; previous experience with research and/or engagement; etc.*

- 1. ***Initial ideas about patient partners’ potential roles/responsibilities within this study***

Here is a brief summary of our study that may help you answer the questions that come next *[below are some possible key points to include in the summary]*

Our study aims to *[please use straightforward language and complete ahead of sharing the form with a patient partner – for example, “… determine whether adult women’s physical activity levels are associated with mental well-being.]*

We propose to meet this aim through *[provide a straightforward description of your study design, including a high-level overview of what data will be collected and how – for example, “… a cross-sectional study, meaning that we will collect participant data at a single point in time. We will measure participants’ physical activity by asking them to wear a device called an accelerometer for a week, and mental well-being and background (sociodemographic) characteristics through questionnaires that they will fill out at a single data collection session at our research center.]*

We have already/we are in the stages of *[what work has already been done of the study – for example, “We have already received grant funding to support this study.”]*.

During our research partnership, the study will be going through the following stages of the research cycle *[list stage(s) and provide straightforward example(s) of what the stage(s) involve(s) if this is potentially unclear (using bullet points will make the information easiest to follow across many stages) – for example: finalizing our study plan; recruitment – identifying and approaching individuals to participate in the study (based on our established protocol); data collection; data analysis; data synthesis (making sense of our findings; knowledge translation (formally writing up our findings and sharing them through journal publications, reports, and presentations)]*

- 1. ***Views on the patient partner role***

1. What does the group see as the purpose and/or value of patient partners (in general and/or specific to this study):

1. What does the group see as the general roles and/or responsibilities of patient partner(s) (in general and/or specific to this study):

1. What does the group see as **academic researchers’** roles and/or responsibilities when engaging patient partners:

Based on the brief description of our study, what are the group’s initial ideas about the potential roles or responsibilities of this study’s patient partner(s)? Are there any potential barriers that could prevent patient partners from engaging, or supports that that may require to fully engage in these roles and responsibilities:
