# Supplementary material for: Exploring patient and caregiver perceptions of the meaning of the patient partner role: a qualitative study
Source: Res Involv Engagem. 2023 Nov 28;9:106. doi: 10.1186/s40900-023-00511-9 (PMC10683322; doi:10.1186/s40900-023-00511-9)
Supplement: Supplementary file 3 — Additional file 3. GRIPP2 short form checklist. [file 40900_2023_511_MOESM3_ESM.docx]

Table 2

GRIPP2 short form

| **Section and topic** | **Item** | **Reported on page No** |
| --- | --- | --- |
| 1: Aim | Report the aim of PPI in the study | 120-129 |
| 2: Methods | Provide a clear description of the methods used for PPI in the study | 189-191 |
| 3: Study results | Outcomes—Report the results of PPI in the study, including both positive and negative outcomes | 192-202 |
| 4: Discussion and conclusions | Outcomes—Comment on the extent to which PPI influenced the study overall. Describe positive and negative effects | 192-202 |
| 5: Reflections/critical perspective | Comment critically on the study, reflecting on the things that went well and those that did not, so others can learn from this experience | 540-559 |

PPI=patient and public involvement
