# Supplementary material for: Exploring patient and caregiver perceptions of the meaning of the patient partner role: a qualitative study
Source: Res Involv Engagem. 2023 Nov 28;9:106. doi: 10.1186/s40900-023-00511-9 (PMC10683322; doi:10.1186/s40900-023-00511-9)
Supplement: Supplementary file 4 — Additional file 4. Semi-structured interview guide. [file 40900_2023_511_MOESM4_ESM.pdf]

Participant ID: ______________________ Interviewer initials:__________

Semi-structured interview guide

Preamble

Thank you so much for speaking with us today and your interest in contributing to our research. Before we begin, we want to check with you about the consent form – do you have questions about it or questions about anything else?

The purpose of this study is to hear about the experiences of patient partners of SPOR) funded studies. By patient partner, we mean a person with lived experience of a health issue, or their informal caregiver, that has become involved in a research project in a role outside of a study participant. We're going to ask you questions to help us learn about how you see your role and your experiences within the role of patient partner. Please remember that anything you say here is confidential, and this is a safe place to talk.

As you may already know, our names are Roger and Anna. We are the patient partner researcher and academic researcher co-leading this project. Since we are really interested in learning about *your* perspectives during this interview, we may ask you to explain some things in as much detail as you can. If we ask you a question that you don't understand or find difficult to answer, please let us know. We may also ask you to clarify things that you have said. This is not because you are not making sense or that we don't understand you. We are just trying to ensure that we are capturing your thoughts and feelings in regards to your experiences and getting as much information as you want to share with us.

You may also notice that we are keeping notes while you talk. We will keep these confidential. These are to help provide context when we read through the interview transcripts in the future and to act as a safeguard in case there are issues with the recording of this interview. Are you still ready and able to participate in this interview today?

Begin recording

This is an interview with ____________________ on (date/time)________________.

We are going to start with asking a bit of background information about you and the SPOR-funded project you worked on. As we are not linking online survey responses with the interviews, many of these questions are similar to the online survey.

1. What province/territory do you live in?

2. What primary community do you represent? For example, patient, caregiver, or both.

3. In what year were you born?

4. What gender do you identify with?

5. How do you best describe your ethnic background? For example, Caucasian or South Asian.

6. What is the highest level of education that you have completed?

7. To re-confirm, the experiences that you are describing today relate to your contributions as a patient partner on SPOR-funded projects?

8. What research phase is this project, or are these projects in? For example, planning phase, recruitment, data collection, etc.

Great. Thank you. The rest of our questions are open-ended, so please feel free to share as much or little as you'd like.

9. **Could you please tell us why did you decide to participate in our study?**

10. Can you please tell us about what your journey has been through healthcare? For example, how has illness impacted your life or your family's life?

- How have your healthcare experiences and/or anything else influenced you in becoming a patient partner?

- How was your illness supported and/or presented burdens to you being a patient partner?

11. We'd now like to hear about who you are as a patient partner and your experiences. Specifically,

- How do you **define** being a patient partner?
- What's **motivated** or driven you to become a patient partner?
  - What do you see as the **purpose** of being a patient partner?
  - What **value** do you see in being a patient partner?
- What do you see as your **roles and responsibilities** (as a patient partner)?
  - Do you feel you had the necessary support to fulfill these responsibilities?

- What would you recommend to help you (or others) be the best patient partner possible in the future?

  - What has and/or hasn’t been helpful towards you being the best research partner you could be?

  - In what ways was and/or wasn’t the environment in which you were a patient partner inclusive?

  - In what ways was and/or wasn’t it a safe environment that promoted honest interactions?

  - In what ways did and/or didn’t you receive adequate support in terms of

    a) training

    b) compensation

    c) education

  - In what ways was/wasn’t there mutual respect?

  - In what ways was/wasn’t there a sense of co-building?

- **What impact do you feel your work as a patient partner has had?**

  a) Do you feel it has had any impact on the research project and/or team?

  b) Do you feel it has had any impact on health research?

  c) Do you feel it has had any impact on health system of the future?

  - Is there any moment during your work as a patient partner that you really felt that you had an impact or not? Do you mind telling us about it?

We'd like to end the interview with four final questions.

12. **When you have finished your involvement with the project, what will you take away and/or what have you taken away?**

13. **In the future, would you be interested in being a patient partner again? Why/why not.**

14. **Under proper circumstances would you be interested in being a patient partner in a full project across its research cycle? Why/why not?**

15. **From your perspective as a patient partner, how do you feel we could get more patient partners involved in research?**

Thank you for your time and for sharing your experiences. **Before we end, is there anything else you'd like us to know about your experiences as a patient partner on SPOR-funded research?**

For your time and interest we'd like to offer you a $25 honorarium in the form of a mailed cheque or an electronic gift card to your choice of Amazon, Superstore, Walmart or Sobeys. If you prefer to receive a mailed cheque, please note that we are required to obtain your Social Insurance Number for the associated paperwork. What is your preferred honorarium?

Honorarium choice (+SIN if cheque)_______________________________________

Interview end time________________________
